# Under which humidity conditions are moss spores released? A comparison between species with perfect and specialized peristomes

**DOI:** 10.1002/ece3.4579

**Published:** 2018-11-08

**Authors:** Florian Zanatta, Alain Vanderpoorten, Lars Hedenäs, Victor Johansson, Jairo Patiño, Niklas Lönnell, Kristoffer Hylander

**Affiliations:** ^1^ Institute of Botany University of Liege Liege Belgium; ^2^ Department of Botany Swedish Museum of Natural History Stockholm Sweden; ^3^ Department of Ecology Swedish University of Agricultural Sciences Uppsala Sweden; ^4^ Department of Ecology, Environment and Plant Sciences Stockholm University Stockholm Sweden; ^5^ Island Ecology and Evolution Research Group Instituto de Productos Naturales and Agrobiologıa (IPNA‐CSIC) La Laguna Spain; ^6^ Department of Environmental Science, Policy and Management University of California Berkeley California; ^7^ Plant Conservation and Biogeography Group, Departamento de Botánica, Ecología y Fisiología Vegetal, Facultad de Ciencias Universidad de La Laguna Tenerife Spain; ^8^ Swedish Species Information Centre Swedish University of Agricultural Sciences Uppsala Sweden

**Keywords:** bryophytes, climatic conditions, dispersal, hygrochastic, peristome, spore, xerochastic

## Abstract

Dispersal is a fundamental biological process that can be divided into three phases: release, transportation, and deposition. Determining the mechanisms of diaspore release is of prime importance to understand under which climatic conditions and at which frequency diaspores are released and transported. In mosses, wherein spore dispersal takes place through the hygroscopic movements of the peristome, the factors enhancing spore release has received little attention. Here, we determine the levels of relative humidity (RH) at which peristome movements are induced, contrasting the response of species with perfect (fully developed) and specialized (reduced) peristomes. All nine investigated species with perfect peristomes displayed a xerochastic behavior, initiating a closing movement from around 50%–65% RH upon increasing humidity and an opening movement from around 90% RH upon drying. Five of the seven species with specialized peristomes exhibited a hygrochastic behavior, initiating an opening movement under increasing RH (from about 80%) and a closing movement upon drying (from about 90%). These differences between species with hygrochastic and xerochastic peristomes suggest that spore dispersal does not randomly occur regardless of the prevailing climate conditions, which can impact their dispersal distances. In species with xerochastic peristomes, the release of spores under decreasing RH can be interpreted as an adaptive mechanism to disperse spores under optimal conditions for long‐distance wind dispersal. In species with hygrochastic peristomes, conversely, the release of spores under wet conditions, which decreases their wind long‐distance dispersal capacities, might be seen as a safe‐site strategy, forcing spores to land in appropriate (micro‐) habitats where their survival is favored. Significant variations were observed in the RH thresholds triggering peristome movements among species, especially in those with hygrochastic peristomes, raising the question of what mechanisms are responsible for such differences.

## 1 INTRODUCTION

Dispersal is a fundamental biological process. Information on dispersal is important for interpreting species distribution patterns and predicting future dynamics and persistence (Travis et al., 2013). Understanding under which climatic conditions diaspores are released and transported is therefore of utmost importance to improve the reliability and accuracy of species distribution models and conservation programs.

In wind‐dispersed species, the dispersal process can be divided into three phases, corresponding to the release, transportation, and deposition of diaspores. Much of the literature focuses on the transportation phase, while the mechanisms triggering diaspore release have been less studied (Greene, 2005; Johansson, Lönnell, Rannik, Sundberg, & Hylander, 2016; Kuparinen, 2006). In particular, the mode and tempo of diaspore release in wind‐dispersed species are largely unknown. Dispersal models therefore often assume random diaspore release in relation to environmental conditions, such as wind speed and humidity (Kuparinen, Markkanen, Riikonen, & Vesala, 2007; Tackenberg, 2003). For many species, however, this assumption is not true (Borger, Renton, Riethmuller, & Hashem, 2012; Johansson, Lönnell, Sundberg, & Hylander, 2014; Pazos, Greene, Katul, Bertiller, & Soons, 2013; Savage, Barbetti, MacLeod, Salam, & Renton, 2012; Skarpaas, Auhl, & Shea, 2006), and the conditions under which diaspores are released may ultimately strongly affect their dispersal distances (Johansson et al., 2016; Savage et al., 2012; Schippers & Jongejans, 2005; Soons & Bullock, 2008).

In most wind‐dispersed species, diaspore release and transportation typically take place under dry conditions promoting the segregation of the diaspores and maximizing dispersal chances (Soons & Bullock, 2008). Diaspores may, however, be released under wet conditions when water is a dispersal vector (Oudtshoorn & Rooyen, 1999). Dispersal under humid conditions could also be selected for if diaspore germination under favorable conditions is a stronger constraint than dispersal distance, such as in arid environments with erratic rains (Hegazy & Kabiel, 2007; Parolin, 2006). Dispersal during wet conditions can therefore be seen as a safe‐site strategy in patchy environments because diaspores are forced to land within the patch in the appropriate (micro‐)environments where their survival is favored (Pufal & Garnock‐Jones, 2010).

**Figure 1:**
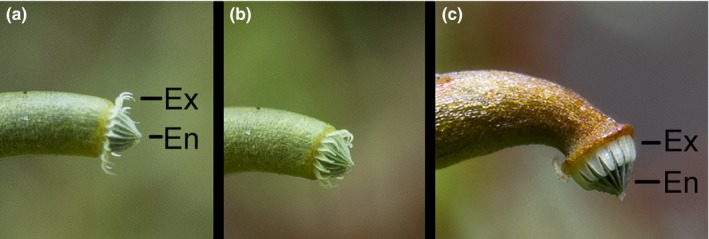
Open (a), closing (b), and closed (c) peristome in *Herzogiella seligeri*. Note that only the exostome (Ex) is hygroscopic, bending toward the endostome (En) when air humidity increases. Field photographs by l. Hedenäs

In bryophytes, spore release is enhanced by the hygroscopic movements (in response to moisture) of elaters or pseudo‐elaters in liverworts and hornworts, respectively, and of the peristome in mosses. The peristome, a unique attribute of mosses that is present in most species, consists of one or two concentric rings of teeth, which are exposed following the loss of the operculum (Goffinet, Buck, & Shaw, 2008). When two rings are present, these form an endostome and an exostome. In peristomes with two rings, only the exostome exhibits hygroscopic movements. The exostome is composed of two radial columns of plates, an inner and an outer set. As water evaporates, the thicker walls shrink and the collective movement along the columns results in the tooth bending toward that side, allowing for hygroscopic movements in response to changes in air humidity (Figure 1). The peristome movements are essential to regulate the dispersal of spores and play an active role in closing and opening the mouth of the capsule depending on variation in air humidity and vibrations caused by wind turbulence (Johansson et al., 2016, 2014 ; Lönnell et al., 2015).

Moss peristomes can be classified into two main categories, namely “perfect” and “specialized” peristomes. The teeth of specialized peristomes are narrower, have a thinner inner layer, and are often shorter than in perfect peristomes. The ornamentation of the external part of the exostome also often differs between species with perfect and specialised peristomes (Hedenäs, 2007). In species with a double peristome, the cilia and basal membrane are frequently reduced or absent. Transitions from a perfect to a specialized state have occurred recurrently, suggesting that these transitions, which typically characterize epiphyte species, are adaptive (Huttunen et al., 2012; Olsson et al., 2009).

Perfect peristomes open‐up upon drying and close‐up under increasing relative humidity (hereafter RH), which is referred to as “xerochastic” peristome movements (Pais, 1964, 1967 ; Steinbrinck, 1897). A xerochastic peristome movement is suggested to maximize the segregation of the spore mass and the chances of long‐distance dispersal under favorable wind conditions (Longton & Schuster, 1983; Medina & Estébanez, 2014). Johansson et al. (2016), however, showed that in *Brachythecium rutabulum,* a moss with a perfect peristome, peristome teeth start to open under decreasing RH already at 87% RH and are totally open at 69% RH. These findings suggest that the spore mass and the surrounding environment could be still quite moist when the capsule starts to open and clouds of spores are thrown out by the moving outer peristome teeth. The contrasting pattern of a peristome that opens up under humid conditions is called “hygrochastic” (Pais, 1964, 1967 ; Steinbrinck, 1897). It has been hypothesized that peristomial reduction would be an adaptation to hygrochasy (Medina & Estébanez, 2014), favoring short‐distance dispersal (Hedenäs, 2012; Vitt, 1981) and representing a safe‐site strategy in species from patchy and dynamic habitats (Medina & Estébanez, 2014). However, there is no systematic evaluation of this hypothesis and quantification of the RH levels that trigger peristome movements has not been performed yet.

In the present paper, we test the existence of distinct patterns in peristome movements (and thus spore release) by measuring the RH thresholds for peristome movements in species with perfect and specialized peristomes. We first aim at confirming that the opening of the peristome under decreasing humidity is a systematic feature among perfect peristomes and that specialized peristomes instead open‐up under increasingly humid conditions (*H*1). Since the reduction in peristomes represents independent evolutionary events, and also might differ in the degree of specialization, we hypothesize that there would be a larger variation in the hygroscopic movements between species with specialized than those that have perfect peristomes (*H*2).

## 2 MATERIAL AND METHODS

We selected nine (*Brachytheciastrum velutinum, Brachythecium rutabulum, Herzogiella seligeri, Amblystegium serpens, Drepanocladus polygamus, Rhytidiadelphus loreus, Hookeria lucens, Thamnobryum alopecurum, and Plagiothecium undulatum*) and seven (*Anomodon viticulosus, Homalothecium sericeum, Leucodon sciuroides, Neckera pennata, Pylaisia polyantha, Orthothecium rufescens, and Pseudoamblystegium subtile*) pleurocarpous mosses with perfect and specialized peristomes, respectively. The sampling strategy ensured that the species, which belong to different genera and to nine families in total, were not closely related to each other (Table 1). For each species, four collections with fertile specimens were sampled, and three sporophytes were studied per collection (Supporting Information Table S1). Since herbarium collections were used, specimens of contrasting ages (3–44 years) were included. Although this factor had to be taken into account in subsequent analyses (see below), the large range of specimen ages investigated returned a robust and conservative idea of the values of RH triggering peristome movements over a wide range of peristome conditions that may also vary in natural conditions due to repeated movements cause by successive episodes of dry and wet climates as well as physical damages.

**Table 1:** Taxonomic sampling, peristome types, and observed peristome movements

Species	Family	Peristome type	Peristome movement
*Amblystegium serpens*	Amblystegiaceae	Perfect	Xerochastic
*Drepanocladus polygamus*	Amblystegiaceae	Perfect	Xerochastic
*Pseudoamblystegium subtile*	Amblystegiaceae	Specialized	Xerochastic
*Anomodon viticulosus*	Anomodontaceae	Specialized	Hygrochastic
*Brachythecium rutabulum*	Brachytheciaceae	Perfect	Xerochastic
*Brachythecium velutinum*	Brachytheciaceae	Perfect	Xerochastic
*Homalothecium sericeum*	Brachytheciaceae	Specialized	Hygrochastic
*Hookeria lucens*	Hookeriaceae	Perfect	Xerochastic
*Rhytidiadelphus loreus*	Hylocomiaceae	Perfect	Xerochastic
*Pylaisia polyantha*	Hypnaceae	Specialized	Hygrochastic
*Leucodon sciuroides*	Leucodontaceae	Specialized	Hygrochastic
*Neckera pennata*	Neckeraceae	Specialized	Hygrochastic
*Thamnobryum alopecurum*	Neckeraceae	Perfect	Xerochastic
*Herzogiella seligeri*	Plagiotheciaceae	Perfect	Xerochastic
*Orthothecium rufescens*	Plagiotheciaceae	Specialized	Intermediate
*Plagiothecium undulatum*	Plagiotheciaceae	Perfect	Xerochastic

**Figure 2:**
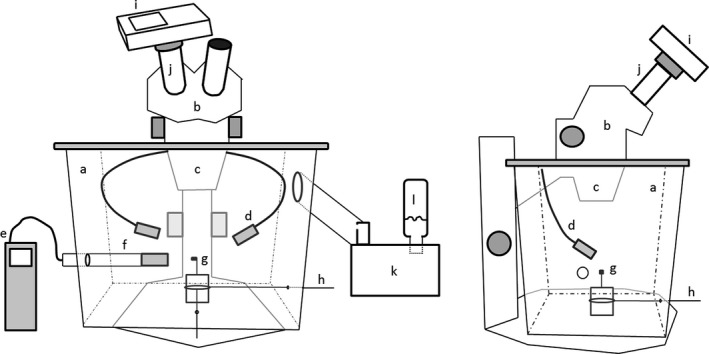
The (a) humidity chamber seen from two angles with a (b) dissecting microscope having its (c) lens and (d) lights immersed in the chamber, a (e) humidity meter having its (f) probe situated 3‐cm sideways of the (g) sporophyte, whose position can be adjusted horizontally by (h) adjustment sticks, and a (i) digital camera that can film the sporophyte through the (j) ocular of the dissecting microscope. Humidity is controlled with an (k) air humidifier (only seen in the left figure) receiving water from a (l) plastic bottle (reproduced with permission from Johansson et al., 2016)

To study peristome movements in relation to humidity, we used a dissecting microscope having its lens immersed into a plastic humidity chamber (Figure 2), designed by Johansson et al. (2016). The experiment took place at room temperature (20ºC) and air humidity (about 30%) in the herbarium of the Natural History Museum of Stockholm. Humidity was regulated in the chamber with a Beurer LB12 ultrasonic humidifier (Beurer, Ulm, Germany) and recorded using the Swema 3,000 hygrometer with the HygroClip2 HC2‐S probe (Swema, Farsta, Sweden) located 3‐cm sideways of the sporophyte. RH was first progressively increased at a rate of about 1% every 1–2 s by adjusting the valve of the humidifier, and decreased at the same rate by opening the valve between the outside (thus at about 30% in the herbarium room) and the chamber. This allowed us to identify the range of RH triggering the first peristome movements upon successively increasing (from 30% to 97%) and decreasing (from 97% to 30%) RH. Having identified this targeted range, a more precise measurement was made by slowly increasing or decreasing RH at a slower rate of about 1% every 5 s. Because we focused on the dynamical response of peristomes to variations of RH, we did not implement here a range of fixed, stable RH conditions using, for instance, sulfuric acid solution at different concentrations (e.g., Oriana & Scatena, 2009), potentially resulting in uncertainties of the actual RH conditions triggering peristome movements. The comparatively low levels of variation observed among measurements made on three capsules independently (Supporting Information Table S1) suggest, however, that our observations are repeatable.

As a first step to the actual measurements, and because we used herbarium specimens whose peristomes have been immobile for long periods, we initiated the experiment by submitting each capsule to a series of three cycles of changes in RH between 30% and 97%. Starting at a relative humidity of 30%, each capsule was subsequently exposed to an increase in RH to 97%, and then to a decrease in RH back to 30%, measuring the RH levels from which peristome movements started in each case. The direction of the movement (opening/closing) was determined qualitatively by observing whether the peristome teeth are bent inwards or outwards. Based on the general pattern of opening or closing under increasing and decreasing condition, we assessed if a species had a xerochastic, hygrochastic, or “intermediate” (when we observed peristome movements but could not determine the general direction of the movement, see below) peristome (*H*1).

To test *H*2, we determined whether species differences accounted for variation in air humidity thresholds of peristome closing and opening in species with specialized and perfect peristomes. These data were homoscedastic and exhibited a normal distribution after arcsine‐transformation in all but one case according to the Goldfeld–Quandt and Shapiro–Wilk tests, respectively. We first determined whether specimen age impacted on the response of the peristomes to variations in RH by computing, for each species upon increasing and decreasing RH, successively, the correlation coefficient between the RH, at which the movement was observed, and specimen age. An analysis of covariance (ANCOVA) was then performed to determine, when RH humidity was increased and decreased separately, whether there were significant differences among species while controlling for the age of the specimen. This analysis was followed by Tukey's HSD grouping tests to identify groups of species with significantly different responses to RH with the R package “agricolae” version 1.2.8 (de Meniburu, 2017). All data were analyzed using R version 3.4.3 (R Development Core Team, 2015).

## 3 RESULTS

**Figure 3:**
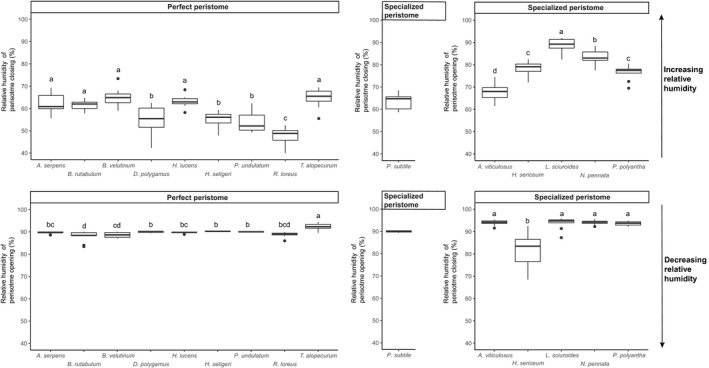
Box‐plots of air humidity levels from which peristome movements were observed at varying air humidity levels in pleurocarpous mosses. Closing and opening movements, respectively, were observed in perfect (left) and specialized (right) peristomes when increasing relative humidity (top) from 30% to 97%. Closing and opening movements, respectively, were observed in specialized (right) and perfect (left) peristomes under decreasing relative humidity conditions (bottom) from 30% to 97%. Differences in opening and closing thresholds among species with perfect and specialized peristomes, respectively, are evidenced by the Tukey's HSD grouping tests. Letters identify groups of species with nonsignificantly different thresholds. See Table 1 for species name abbreviations

All nine species with perfect peristomes displayed a xerochastic behavior with a consistent pattern of peristome closing (i.e., inward movement of the exostome teeth toward the endostome) when the moisture increased and an opening (i.e., backward movement of the exostome teeth away from the endostome) when RH decreased (Figure 3). Typically, the closing of the peristome started at around 50%–65% RH upon moistening and the opening started at around 90% RH upon drying.

Five of the seven species with specialized peristomes exhibited the hypothesized hygrochastic behavior with an opening of the peristome under increasing RH and a closing under decreased RH (Figure 3). Hygrochastic peristomes started to open‐up from about 80% RH onwards and started to close when RH decreased below about 90% (Figure3). However, *P. subtile* displayed a typical xerochastic behavior, whereas it was impossible to determine whether the movements observed in *O. rufescens* upon varying air humidity could be classified as a clear closing or opening. Consequently, *O. rufescens* could thus not be classified as either xerochastic or hygrochastic.

Significant correlation coefficients were observed between specimen age and the RH at which peristome movements were observed, both upon increasing and decreasing RH, in three out of the 16 species investigated (Supporting Information Figure S1). In *D. polygamus*, the closing and opening of the xerochastic peristome occurred at lower RH in recent than in old specimens upon increasing and decreasing RH, respectively. In *H. sericeum* and *L. sciuroides,* the opening and the closing of the peristome occurred at lower RH in old than in recent specimens upon increasing and decreasing RH, respectively. In all cases, the variation in peristome response due to age differences was of a few percent of RH.

There was a trend for a higher variation in opening and closing humidity thresholds in species with specialized peristomes as compared to species with perfect peristomes (Figure 3). Despite this, there was a significant difference in these thresholds among species for both specialized and perfect peristomes (Figure 3) while controlling for the mostly significant effect of specimen age, although the significance of the interaction between species and specimen age potentially blurred the signal of the former (Table 2).

**Table 2:** Analysis of covariance on the variation of the relative humidity levels triggering peristome movement upon increasing and decreasing air relative humidity among moss species with a xerochastic and hygrochastic peristome while controlling for specimen age

	*df*	MS	*F*	*p* value
Xerochastic peristomes				
Increasing RH				
Collection date	1	0.046	34.022	**<0.001**
Species	8	0.038	28.139	**<0.001**
Interaction term	8	0.006	4.403	**<0.001**
Error	90	0.001		
Decreasing RH				
Collection date	1	>0.001	0.690	0.408
Species	8	0.005	19.132	**<0.001**
Interaction term	8	>0.001	0.827	0.585
Error	90	>0.001		
Hygrochastic peristomes				
Increasing RH				
Collection date	1	0.148	100.210	**<0.001**
Species	4	0.083	56.381	**<0.001**
Interaction term	4	0.002	1.534	0.206
Error	52	0.001		
Decreasing RH				
Collection date	1	0.009	6.989	**0.010**
Species	4	0.118	88.584	**<0.001**
Interaction term	4	0.004	2.676	**0.041**
Error	52	0.001		

Note

*df*, degrees of freedom; MS, mean sum of squares; *F* statistic and associated *p* value, highlighted in bold when significant at the *α* = 0.05 level.

## 4 DISCUSSION

Although Johansson et al. (2016) did not find any significant differences between fresh and herbarium specimens in *B. rutabulum,* our results show that part of the variation of the RH level triggering peristome movements was caused by age differences among specimens. Indeed, significant differences in the RH, at which peristome movements were observed, were found between old and recent specimens in three out of the 16 investigated species. While the response of peristome teeth to variation of RH was delayed for the closing movement in old specimens, the opening of the peristome of more recent specimens was, conversely, delayed as compared to that of old specimens. This suggests that structural damages to the peristomes of old specimens may affect the functioning of the peristome, but not necessarily toward a delayed response. The fact, that such differences were observed in a few species, and that the differences due to specimen age were of a few percent of RH only, suggests that the use of herbarium specimens does not globally affect our conclusions on the response of different kinds of peristomes to variation in RH.

In agreement with our primary hypothesis (*H*1), perfect peristomes consistently exhibited a typical xerochastic behavior, whereas specialized peristomes were mostly hygrochastic. The only exception was for *P. subtile*, wherein the exostome teeth are, however, not much reduced as compared to the other investigated species with specialized peristomes. *O. rufescens,* for which we could not determine the type of movements, should be termed intermediate following Pais (1964). These differences between hygrochastic and xerochastic peristomes confirm previous studies demonstrating that diaspores dispersal does not randomly occur regardless of the prevailing climate conditions, which can impact their dispersal distances (Savage et al., 2012; Schippers & Jongejans, 2005; Soons & Bullock, 2008), questioning the use of dispersal models that assume random diaspore release in relation to environmental conditions (Kuparinen et al., 2007; Tackenberg, 2003).

The observation that the opening of xerochastic peristomes starts already at quite high moisture levels, however, challenges the notion that the spores of the species of this functional group are released under dry conditions (Longton & Schuster, 1983) and confirm the idea that xerochastic peristomes release their spores under decreasing, but still relatively high air humidity conditions (Johansson et al., 2016). Johansson et al. (2016) interpreted the behavior of xerochastic peristomes as an adaptive mechanism favoring the release of spores in the morning when the heating from the sun creates upward air movements, which can carry away spore over long distances.

In species with specialized peristomes, conversely, spores are released under increasing wet conditions and, therefore, decreasing chances for long‐distance dispersal by wind. Although peristome specialization often prevails in species from patchy habitats, and in particular, epiphytes, wherein the release of spores under wet conditions seems counter intuitive as epiphyte communities need to track patches of suitable trees in a dynamic landscape (Snäll, Ehrlén, & Rydin, 2005; Snäll, Ribeiro, & Rydin, 2003), two lines of evidence suggest that dispersal ability in epiphytes is counter‐selected. First, epiphytic communities are significantly spatially structured, so that epiphyte species composition is more similar among trees located near to each other than among trees distant from each other (Löbel, Snäll, & Rydin, 2006a, 2006b; Patiño, Gomez‐Rodriguez, Pupo‐Correia, Sequeira, & Vanderpoorten, 2018; Wagner, Mendieta‐Leiva, & Zotz, 2015). Second, epiphyte colonization probability decreases with increasing isolation, as shown by analyses of spatial distributions (Johansson, Ranius, & Snäll, 2012; Ruete, Fritz, & Snäll, 2014; Snäll et al., 2005) and evidence for significant isolation‐by‐distance patterns inferred from spatial genetic structures at short spatial scales (Patiño et al., 2013; Snäll, Fogelqvist, Ribeiro, & Lascoux, 2004). In this context, the release of spores under wet conditions, which decreases their long‐distance dispersal capacities, might be seen as a safe‐site strategy, forcing spores to land in appropriate (micro‐) habitats where their survival is favored (Medina & Estébanez, 2014). In fact, high levels of humidity are thought to enhance the probability that spores adhere to vertical boles and branches (Hedenäs, 2002) and are critical for initial establishment stages in such harsh environments as bark (Wiklund & Rydin, 2004). Our findings therefore reinforce the idea that establishment is a stronger evolutionary bottleneck than dispersal capacity (Johansson et al., 2016).

In agreement with our second hypothesis (*H*2), the variation in peristome movement was larger in the group of specialized peristomes, including one species with an “intermediate” behavior (*O. rufescens*) and one species with a xerochastic behavior (*P. subtile*). Despite that perfect peristomes exhibit lower interspecific differences than specialized ones, which vary in their degree of specialization, there were, however, significant interspecific differences in the response of the peristome to variations of RH also within the group of perfect peristomes. Although significant interactions between the factors specimen age and species potentially blurred the signal of the latter, the presence of interspecific differences in the response within both specialized and perfect peristomes point to the role of structural variations beyond peristome reductions. The similar levels of relative humidity triggering the opening of the peristome under decreasing and increasing humidity conditions in xerochastic and hygrochastic peristomes, respectively, is, however, suggestive of the existence of functional constraints. Understanding the mechanisms behind such differences, discussed by Mueller and Neumann (1988), requires further experimental studies. One the one hand, the number and distribution of stomata, which play an active role in the desiccation of the sporophyte (Duckett & Pressel, 2018), may contribute to variations of the relative humidity within the capsule, thereby potentially affecting peristome movements. On the other hand, variation in peristome ornamentation, whose function is still poorly understood, might play a key role in explaining the movements of peristomes depending on relative air humidity, and hence, in controlling spore release as a function of local climate conditions.

## CONFLICT OF INTEREST

None declared.

## AUTHORS’ CONTRIBUTION

Author contributions: LH, VJ, NL, and KH conceived the project. FZ and VJ collected the data. J.P and AV performed the statistical analyses. All the authors contributed to the writing of the manuscript.

## DATA ACCESSIBILITY

The data used in the present study are displayed in Supporting Information Table S1.
