# Supplementary figures and images for: Under which humidity conditions are moss spores released? A comparison between species with perfect and specialized peristomes
Source: Ecol Evol. 2018 Nov 8;8(23):11484–91. doi: 10.1002/ece3.4579 (PMC6303758; doi:10.1002/ece3.4579)

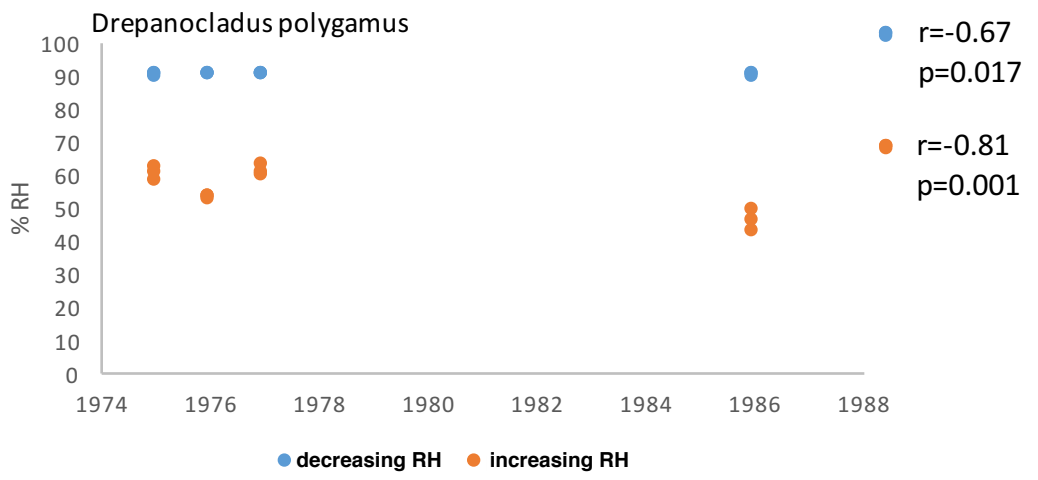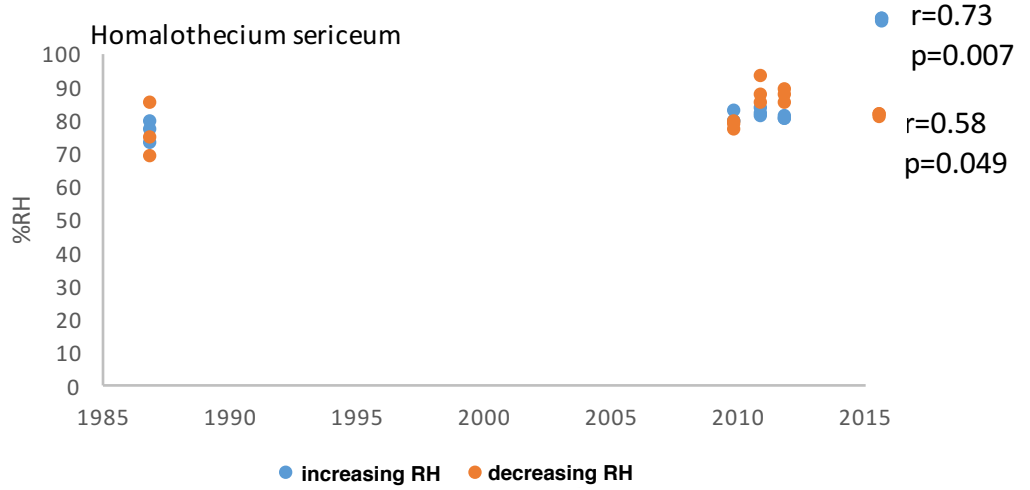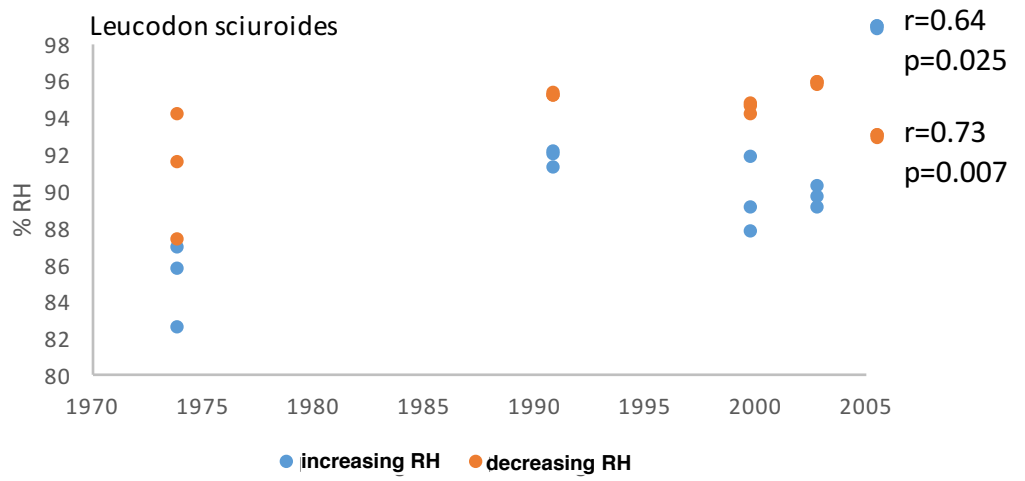

Supplement: Supplementary file 1 [file ECE3-8-11484-s001.pdf]
