# Supplementary material for: Under which humidity conditions are moss spores released? A comparison between species with perfect and specialized peristomes
Source: Ecol Evol. 2018 Nov 8;8(23):11484–91. doi: 10.1002/ece3.4579 (PMC6303758; doi:10.1002/ece3.4579)
Supplement: Supplementary file 2 [file ECE3-8-11484-s002.docx]

**Table S1.** List of the herbarium specimens [reference number of the Stockholm herbarium, S#, collecting date) used for the study of peristome movements, percentage air humidity from which xerochastic peristomes initiated the closing (C) and opening (O) movements under increasing and decreasing RH levels, respectively, and percentage air humidity from which hygrochastic peristomes initiated the opening (O) and closing (C) movements under increasing and decreasing RH levels, respectively.

| S# | Date | | |  | | |  | | |
| --- | --- | --- | --- | --- | --- | --- | --- | --- | --- |
| **Xerochastic peristomes** |  | | | C | | | O | | |
| *Brachytheciastrum velutinum* | | | | | |  | | |  |
| B111561 | 5/1/2006 | | | 68.0 | | | 89.9 | | |
|  |  | | | 66.0 | | | 87.6 | | |
|  |  | | | 66.0 | | | 87.0 | | |
| B10559 | 5/1/1985 | | | 65.0 | | | 87.7 | | |
|  |  | | | 68.0 | | | 89.8 | | |
|  |  | | | 63.0 | | | 89.7 | | |
| B179031 | 5/21/2010 | | | 64.7 | | | 87.2 | | |
|  |  | | | 60.1 | | | 90.1 | | |
|  |  | | | 63.2 | | | 87.3 | | |
| B160300 | 3/22/2009 | | | 73.4 | | | 88.1 | | |
|  |  | | | 59.0 | | | 89.3 | | |
|  |  | | | 61.5 | | | 89.2 | | |
| B200811 | 9/10/2013 | | | 68.0 | | | 89.9 | | |
|  |  | | | 66.0 | | | 87.6 | | |
|  |  | | | 66.0 | | | 87.0 | | |
| B183049 | 4/23/2011 | | | 65.0 | | | 87.7 | | |
|  |  | | | 68.0 | | | 89.8 | | |
|  |  | | | 63.0 | | | 89.7 | | |
| B97616 | 5/12/1986 | | | 64.7 | | | 87.2 | | |
|  |  | | | 60.1 | | | 90.1 | | |
|  |  | | | 63.2 | | | 87.3 | | |
| B70334 | 5/5/1973 | | | 73.4 | | | 88.1 | | |
|  |  | | | 59.0 | | | 89.3 | | |
|  |  | | | 61.5 | | | 89.2 | | |
| *Herzogiella seligeri* |  | | |  | | |  | | |
| B177625 | 9/1/2010 | | | 56.1 | | | 90.3 | | |
|  |  | | | 52.0 | | | 90.3 | | |
|  |  | | | 51.2 | | | 90.3 | | |
| B165625 | 9/5/2009 | | | 59.4 | | | 90.3 | | |
|  |  | | | 54.6 | | | 90.3 | | |
|  |  | | | 47.9 | | | 90.3 | | |
| B48763 | 10/19/1988 | | | 56.5 | | | 90.1 | | |
|  |  | | | 57.7 | | | 90.2 | | |
|  |  | | | 58.6 | | | 89.9 | | |
| B202534 | 9/30/2010 | | | 54.0 | | | 90.0 | | |
|  |  | | | 57.3 | | | 90.1 | | |
|  |  | | | 56.0 | | | 90.2 | | |
| *Plagiothecium undulatum* |  | | |  | | |  | | |
| B165650 | 9/19/2009 | | | 52.3 | | | 89.8 | | |
|  |  | | | 50.3 | | | 90.0 | | |
|  |  | | | 50.1 | | | 90.2 | | |
| B94273 | 7/17/2004 | | | 60.4 | | | 90.0 | | |
|  |  | | | 60.5 | | | 90.2 | | |
|  |  | | | 62.3 | | | 90.2 | | |
| B8170 | 9/3/1998 | | | 52.0 | | | 89.9 | | |
|  |  | | | 54.8 | | | 89.7 | | |
|  |  | | | 55.9 | | | 90.2 | | |
| B48751 | 10/19/1988 | | | 49.3 | | | 90.0 | | |
|  |  | | | 50.3 | | | 90.3 | | |
|  |  | | | 51.9 | | | 89.7 | | |
| *Amblystegium serpens* |  | | |  | | |  | | |
| B183595 | 6/6/2011 | | | 60.3 | | | 89.7 | | |
|  |  | | | 61.2 | | | 90.0 | | |
|  |  | | | 67.5 | | | 89.7 | | |
| B99802 | 6/30/2004 | | | 65.3 | | | 89.7 | | |
|  |  | | | 67.4 | | | 88.6 | | |
|  |  | | | 69.3 | | | 88.7 | | |
| B179336 | 9/2/2010 | | | 55.6 | | | 90.0 | | |
|  |  | | | 60.5 | | | 90.1 | | |
|  |  | | | 58.6 | | | 89.7 | | |
| B201872 | 10/15/2008 | | | 62.9 | | | 90.0 | | |
|  |  | | | 58.9 | | | 89.7 | | |
|  |  | | | 60.3 | | | 89.7 | | |
| *Drepanocladus polygamus* |  | | |  | | |  | | |
| B202460 | 8/3/1977 | | | 59.2 | | | 90.2 | | |
|  |  | | | 60.1 | | | 90.2 | | |
|  |  | | | 62.5 | | | 89.8 | | |
| B202461 | 10/22/1975 | | | 60.4 | | | 90.2 | | |
|  |  | | | 61.5 | | | 89.6 | | |
|  |  | | | 57.7 | | | 90.2 | | |
| B210282 | 7/6/1986 | | | 49.2 | | | 89.4 | | |
|  |  | | | 45.9 | | | 89.7 | | |
|  |  | | | 42.3 | | | 89.8 | | |
| B210283 | 7/10/1976 | | | 52.3 | | | 90.1 | | |
|  |  | | | 53.2 | | | 90.2 | | |
|  |  | | | 53.0 | | | 90.2 | | |
| *Pseudoamblystegium subtile* |  | | |  | | |  | | |
| B178608 | 9/1/2010 | | | 64.5 | | | 90.3 | | |
|  |  | | | 65.6 | | | 90.3 | | |
|  |  | | | 65.1 | | | 90.0 | | |
| B201876 | 9/6/2007 | | | 60.5 | | | 90.3 | | |
|  |  | | | 61.2 | | | 90.2 | | |
|  |  | | | 65.0 | | | 90.3 | | |
| B201881 | 10/15/2008 | | | 58.7 | | | 89.7 | | |
|  |  | | | 59.3 | | | 89.7 | | |
|  |  | | | 59.0 | | | 89.7 | | |
| B76242 | 12/1/2002 | | | 65.7 | | | 89.3 | | |
|  |  | | | 68.2 | | | 89.7 | | |
|  |  | | | 68.5 | | | 90.2 | | |
| *Rhytidiadelphus loreus* |  | | |  | | |  | | |
| B26548 | 7/2/1985 | | | 45.2 | | | 89.9 | | |
|  |  | | | 49.0 | | | 88.5 | | |
|  |  | | | 52.5 | | | 89.1 | | |
| B28367 | 7/21/1990 | | | 52.1 | | | 89.1 | | |
|  |  | | | 45.8 | | | 89.7 | | |
|  |  | | | 45.6 | | | 89.3 | | |
| B108215 | 5/12/1966 | | | 49.5 | | | 86.0 | | |
|  |  | | | 52.1 | | | 89.1 | | |
|  |  | | | 48.6 | | | 88.7 | | |
| B108441 | 11/16/1958 | | | 47.0 | | | 89.8 | | |
|  |  | | | 49.2 | | | 89.1 | | |
|  |  | | | 40.0 | | | 88.2 | | |
| *Hookeria lucens* |  | | |  | | |  | | |
| B211565 | 5/24/1993 | | | 62.4 | | | 88.9 | | |
|  |  | | | 64.2 | | | 89.4 | | |
|  |  | | | 62.8 | | | 89.6 | | |
| B211563 | 5/7/1978 | | | 65.1 | | | 89.7 | | |
|  |  | | | 61.2 | | | 89.9 | | |
|  |  | | | 62.4 | | | 89.9 | | |
| B42902 | 5/26/1994 | | | 58.2 | | | 89.8 | | |
|  |  | | | 63.1 | | | 89.8 | | |
|  |  | | | 61.2 | | | 89.9 | | |
| B203405 | 4/13/2014 | | | 68.4 | | | 89.8 | | |
|  |  | | | 64.4 | | | 89.9 | | |
|  |  | | | 64.6 | | | 89.9 | | |
| *Thamnobryum alopecurum* |  | | |  | | |  | | |
| B9903 | 6/8/1991 | | | 68.5 | | | 91.8 | | |
|  |  | | | 60.5 | | | 94.5 | | |
|  |  | | | 67.5 | | | 94.2 | | |
| B9904 | 6/8/1991 | | | 55.5 | | | 89.6 | | |
|  |  | | | 65.5 | | | 92.1 | | |
|  |  | | | 65.5 | | | 91.1 | | |
| B9923 | 5/30/1991 | | | 66.5 | | | 93.3 | | |
|  |  | | | 63.5 | | | 91.9 | | |
|  |  | | | 69.5 | | | 90.9 | | |
| B9925 | 5/31/1991 | | | 62.5 | | | 93.7 | | |
|  |  | | | 65.5 | | | 92.6 | | |
|  |  | | | 68.5 | | | 92.8 | | |
| **Hygrochastic peristomes** |  | | | **O** | | | **C** | | |
| *Homalothecium sericeum* | |  |  | |  | | |  |  |
| B174820 | 4/28/2010 | | | 81.6 | | | 78.5 | | |
|  |  | | | 78.6 | | | 76.0 | | |
|  |  | | | 78.5 | | | 78.1 | | |
| B192234 | 4/27/2012 | | | 79.6 | | | 88.3 | | |
|  |  | | | 80.1 | | | 87.2 | | |
|  |  | | | 79.4 | | | 84.7 | | |
| B183384 | 5/27/2011 | | | 82.5 | | | 92.5 | | |
|  |  | | | 81.2 | | | 87.1 | | |
|  |  | | | 80.4 | | | 84.5 | | |
| B53428 | 4/25/1987 | | | 78.8 | | | 84.6 | | |
|  |  | | | 76.5 | | | 74.0 | | |
|  |  | | | 72.1 | | | 68.5 | | |
| *Leucodon sciuroides* |  | | |  | | |  | | |
| B102554 | 3/7/1991 | | | 92.0 | | | 95.1 | | |
|  |  | | | 91.2 | | | 95.2 | | |
|  |  | | | 91.8 | | | 95.0 | | |
| B80185 | 4/11/2003 | | | 90.1 | | | 95.7 | | |
|  |  | | | 89.0 | | | 95.6 | | |
|  |  | | | 89.5 | | | 95.8 | | |
| B211823 | 7/2/1974 | | | 85.6 | | | 91.4 | | |
|  |  | | | 82.4 | | | 87.3 | | |
|  |  | | | 86.8 | | | 94.0 | | |
| B44543 | 9/24/2000 | | | 91.7 | | | 94.6 | | |
|  |  | | | 88.9 | | | 94.4 | | |
|  |  | | | 87.7 | | | 94.0 | | |
| *Anomodon viticulosus* |  | | |  | | |  | | |
| B196243 | 3/31/2013 | | | 74.5 | | | 93.1 | | |
|  |  | | | 67.5 | | | 94.0 | | |
|  |  | | | 74.5 | | | 94.1 | | |
| B192604 | 5/20/2012 | | | 67.5 | | | 95.4 | | |
|  |  | | | 69.5 | | | 94.8 | | |
|  |  | | | 68.5 | | | 94.4 | | |
| B193392 | 7/22/2012 | | | 65.5 | | | 95.4 | | |
|  |  | | | 68.5 | | | 94.6 | | |
|  |  | | | 64.5 | | | 94.8 | | |
| B182473 | 4/5/2011 | | | 62.5 | | | 92.5 | | |
|  |  | | | 61.5 | | | 93.9 | | |
|  |  | | | 70.5 | | | 91.6 | | |
| *Neckera pennata* |  | | |  | | |  | | |
| B16956 | 9/14/1999 | | | 77.5 | | | 92.3 | | |
|  |  | | | 78.5 | | | 92.9 | | |
|  |  | | | 80.5 | | | 92.4 | | |
| B16954 | 9/11/1999 | | | 84.5 | | | 94.2 | | |
|  |  | | | 85.5 | | | 95.6 | | |
|  |  | | | 82.5 | | | 95.6 | | |
| B16955 | 9/15/1999 | | | 88.5 | | | 95.1 | | |
|  |  | | | 86.5 | | | 94.4 | | |
|  |  | | | 86.5 | | | 94.4 | | |
| B16953 | 9/11/1999 | | | 82.5 | | | 94.5 | | |
|  |  | | | 83.5 | | | 94.1 | | |
|  |  | | | 82.5 | | | 94.0 | | |
| *Pylaisia polyantha* |  | | |  | | |  | | |
| B205140 | 6/26/2014 | | | 77.5 | | | 92.5 | | |
|  |  | | | 77.5 | | | 92.2 | | |
|  |  | | | 72.5 | | | 94.3 | | |
| B210870 | 4/26/2012 | | | 77.5 | | | 93.1 | | |
|  |  | | | 77.5 | | | 94.7 | | |
|  |  | | | 77.5 | | | 94.1 | | |
| B184639 | 8/15/2011 | | | 72.5 | | | 93.7 | | |
|  |  | | | 77.5 | | | 94.8 | | |
|  |  | | | 80.5 | | | 93.9 | | |
| B210871 | 3/5/2011 | | | 69.5 | | | 93.2 | | |
|  |  | | | 79.5 | | | 92.4 | | |
|  |  | | | 80.5 | | | 94.9 | | |
| **Intermediate peristomes** | | | | |  | | |  |  |
| *Orthothecium rufescens* | |  |  | |  | | |  |  |
| B8173 | 9/4/1998 | | | 75.2 | | | 94.0 | | |
|  |  | | | 72.9 | | | 95.9 | | |
|  |  | | | 73.1 | | | 95.9 | | |
| B197316 | 6/17/2013 | | | 75.4 | | | 92.6 | | |
|  |  | | | 73.0 | | | 95.2 | | |
|  |  | | | 74.2 | | | 94.7 | | |
| B4641 | 7/14/1992 | | | 73.3 | | | 96.3 | | |
|  |  | | | 75.6 | | | 96.1 | | |
|  |  | | | 72.1 | | | 95.8 | | |
| B6816 | 8/6/1994 | | | 72.5 | | | 95.1 | | |
|  |  | | | 76.0 | | | 95.1 | | |
|  |  | | | 77.3 | | | 95.2 | | |
